# Human T2R38 Bitter Taste Receptor Expression in Resting and Activated Lymphocytes

**DOI:** 10.3389/fimmu.2018.02949

**Published:** 2018-12-11

**Authors:** Hoai T. T. Tran, Corinna Herz, Patrick Ruf, Rebecca Stetter, Evelyn Lamy

**Affiliations:** ^1^Molecular Preventive Medicine, Institute for Infection Prevention and Hospital Epidemiology, University Medical Center and Faculty of Medicine, University of Freiburg, Freiburg, Germany; ^2^Pharmaceutical Bioinformatics, Institute of Pharmaceutical Sciences, Albert-Ludwigs-University, Freiburg, Germany

**Keywords:** G protein coupled receptor (GPCR), human bitter taste receptor (T2R), T2R38, hTAS2R38 gene, aging, human T cells

## Abstract

The human G-protein-coupled bitter taste receptor T2R38 has recently been demonstrated to be expressed on peripheral blood neutrophils, monocytes and lymphocytes. To further define a potential contribution of the T2R38 receptor in adaptive immune response, the objective of this study was to analyze its expression in resting and activated lymphocytes and T cell subpopulations. Freshly isolated PBMC from healthy donors were used for expression analysis by flow cytometry. Quantum™ MESF beads were applied for quantification in absolute fluorescence units. Activation methods of T cells were anti-CD3/CD28, phytohaemagglutinin (PHA) or phorbol 12-myristate 13-acetate (PMA) together with ionomycin. Lymphocytes from young donors expressed higher levels of T2R38 compared to the elderly. CD3+ T cells expressed higher levels that CD19+ B cells. Receptor expression followed T cell activation with an upregulation within 24 h and a peak at 72 h. Higher levels of T2R38 were produced in lymphocytes by stimulation with anti-CD3/CD28 compared to PHA or PMA/ionomycin. Both subpopulations of CD4+ as well as CD8+ T cells were found to express the T2R38 receptor; this was higher in CD4+ than CD8+ cells; the amount of T2R38 in central and effector memory cells was higher as compared to naïve cells, although this was not statistically significant for CD8+ cells without prior activation by anti-CD3/CD28. Upon treatment of PBMC with the natural T2R38 agonist goitrin Calcium flux was activated in the lymphocyte population with functional T2R38 receptor at >20 μM which was completely blocked by phospholipase Cβ-2 inhibitor U73211. Further, goitrin selectively inhibited TNF-alpha secretion in PBMC with functional T2R38. This quantitative analysis of T2R38 expression in distinct PBMC subsets may provide a basis for understanding the significance of bitter compounds in immune modulation. Whether these findings can have implications for the treatment of inflammatory and immunologic disorders by bitter tasting pharmaceuticals or foods needs further investigation.

## Introduction

The primary task of tasting bitter is thought to serve a sentinel function to prevent from toxin ingestion (1). The sensors for bitter substances are G-protein coupled receptors (GPCRs) originally identified in type II taste receptor cells in the taste bud of the oral cavity (2). In humans, 25 different bitter taste receptors (T2Rs) are known to be expressed (3). One of them, the antithyroid-toxin receptor T2R38 responds to compounds which contain a thiourea (N-C = S) moiety such as goitrin or its precursor progoitrin as well as compounds containing an isothiocyanate (N = C = S) moiety (4). These are mainly, but not exclusively bitter tasting compounds that are widely distributed in plants of the family *Brassicaceae*. Meanwhile, an extra gustatory expression of the T2R38 receptor has been reported. Its physiological relevance in tissue outside the gustatory systems is, however, still elusive. T2R38 receptor expression was found in epithelial cells of the airways (5), human placenta (6) or the colon (7), and most interestingly here in peripheral leucocytes (neutrophils, monocytes, and lymphocytes) (8). So far, Carey et al. (9) concluded that cilia are needed for T2R38 function in respiratory innate immunity, and a previous study addressed the role of T2R38 for local host defense proposing that the T2R38 could be an important mediator of sinonasal epithelial defense (10). Data from 5 suggest a role for T2R38 in chronic rhinosinusitis and bacterial detection. On neutrophils, T2R38 was proposed to serve as a receptor for quorum sensing molecules, which are produced by *Pseudomonas aeruginosa* and other Gram-negative bacteria (8, 10). A recent study supported this idea of T2R38 serving as bacterial detector by identifying several bacterial metabolites as trigger compounds (11). However, Verbeurgt et al. also questioned the results derived earlier with the quorum sensing molecules HSLC4 and HSLC12. In their model system, they could not reproduce the findings published earlier (5) and proposed a potential solvent effect (11). Thus, there is still great need for more confirmatory studies to understand the role of T2R38 in the human immune response. Establishing knowledge about T2R38 expression in resting and activated lymphocytes is important here in order to gain new insights into the potential relevance of this bitter receptor in the adaptive immune response. In the present study, the expression of T2R38 was assessed using human peripheral mononuclear blood cells (PBMC), freshly isolated from young and elderly donors. Besides age, the effects of stimulation on T2R38 expression in lymphocytes and their T cell subsets were investigated. Functional relevance of the receptor for lymphocytes was investigated using goitrin in a Calcium-based assay. Additionally, TNF-alpha secretion was quantified in PBMC with functional and non-functional T2R38 receptor upon goitrin treatment.

## Materials and Methods

### Chemicals

Fetal calf serum (FCS), L-glutamine and phosphate buffered saline (PBS, without Ca and Mg), penicillin-streptomycin (P/S) solution and RPMI-1640 were from Life Technologies (Darmstadt, Germany). Hank's Balanced Salt Solution (HBSS) was from PAA Laboratories GmBH (Coelbe, Germany). Dimethyl sulfoxide (DMSO; purity >99 %) was purchased from Applichem GmbH (Darmstadt, Germany). DL-Goitrin was purchased from Santa Cruz Biotechnology (Heidelberg, Germany). Nuclease free water was from Qiagen (Hilden, Germany). The primary antibody, anti-T2R38 (rabbit IgG ab130503, to C-terminus), the rabbit IgG isotype control and the fluorophore-labeled secondary antibodies, anti-rabbit-Alexa 488, were purchased from Abcam (Cambridge, UK). The following primary human antibodies labeled with fluorophore were used for flow cytometry: CD3-APC (clone BW264/56), CD3-PE (clone REA613), CD4-PE (clone 15E8), CD4-APC (clone REA 623), CD8-PE (clone BW135/80), CD8-APC (clone BW135/80), CD45RO-Percp (clone UCHL1), CD62L-APC (Clone 145/15), CD69-PE (Clone FN50), CD11b (clone M1/70.15.11.5), CD14-PE (clone REA599) from Miltenyi Biotec (Bergisch Gladbach, Germany), CD19-APC (clone HIB19) from eBioscience, CD25-Percp (clone M-A251) from Bioledgen. Functional anti-human CD3 (clone OKT3), and CD28 (clone 28.2) were purchased from eBioscience (Frankfurt, Germany). Recombinant human Interleukine-2 (IL-2) and red blood cell lysis solution (10x) was purchased from Miltenyi Biotec. LymphoPrep™ was from Alere Technologies AS (Oslo, Norway). Ionomycin (purity ≥98%) and phorbol 12-myristate 13-acetate (PMA) from Cayman Europe (purity ≥98%, Ann Arbor, Michigan, USA), phytohaemagglutinin (PHA-L) was from Sigma Aldrich (Taufkirchen, Germany), U73122 was purchased from Enzo Life Sciences (Lörrach, Germany). Fluo-4-AM and Pluronic™ F-127 was from Thermo Fisher Scientific GmBH (Darmstadt, Germany).

### Human PBMC

Human PBMC were isolated from the fresh peripheral blood of volunteers at the University Medical Center in Freiburg, Germany. Written informed consent was given by all subjects and the study was approved by the ethics committee of the University of Freiburg (No. 597/14). Blood was collected from 56 volunteers using Li-heparinized vacutainers (Sarstedt, Nümbrecht, Germany) after obtaining written informed consent. The volunteers (male and female, aged between 20–35 and 60–90 years) had a normal BMI, were healthy and non-smokers. Granulocytes were isolated from whole blood by using red blood cell lysis buffer according to the manufacturer's instructions. PBMC were isolated from the blood within 2 h by centrifugation on a LymphoPrep™ gradient (density: 1.077 g/cm^3^) at 1,200 g for 13 min at room temperature, using 50 ml SepMate™ (Grenoble, France) tubes, were then washed twice with PBS, and cell viability and concentration were determined using the trypan blue exclusion test. PBMC were used directly for immunostaining or cultured in RPMI 1640 medium supplemented with 10% heat-inactivated FCS, 2 mM L-glutamine, 100 U/mL penicillin/streptomycin at 37°C in a humidified incubator with a 5% CO_2_/ 95% air atmosphere. Cell activation was done by using functional antibodies to CD3 and CD28, PHA (solvent control: aqua dest), or PMA together with 1 μg/ml ionomycin (solvent control: 0.02% DMSO).

### Flow Cytometric Analysis of T2R38 Expression

Isolated immune cells were stained with an appropriate combination of different surface markers (anti-CD3/4/8-PE or APC, anti-CD25-Percp, anti-CD45Ro-Percp, anti-62L-APC, anti-69-PE for 30 min at 4°C. Cells were fixed and permeabilized using BD Cytofix/CytopermTM Fixation/Permeabilization Kit (BD Biosciences, Heidelberg, Germany) according to the manufacturer's protocol before staining with the primary antibody anti-T2R38 or IgG, conjugated with Alexa Fluor 488 coupled secondary antibodies. T2R38 expression was quantified with a FACSCalibur™ (BD Biosciences, Heidelberg, Germany) using the Quantum™ MESF Alexa Fluor® 488 Kit (Polysciences Europe GmbH, Hirschberg an der Bergstrasse, Germany) according to the manufacturer's protocol. In short, the median fluorescence intensity (MFI) of each sample was recorded using the FlowJo software (Ashland, Oregon, USA). The Quantum MESF beads were run on the same day and at the same fluorescence settings as the stained cell samples to establish a calibration curve relating instrument channel values to standardized fluorescence intensity (MESF) units. Recorded MFI values were then converted to MESF units using the QuickCal program v.2.3 (Polysciences Europe GmbH) according to the protocol. Finally, the delta MESF units (MESF units of anti-T2R38 - MESF units of anti-IgG control) were calculated.

### Quantification of TNF-Alpha Release

PBMC (1 × 10^6^ cells/ml) were cultured in RPMI 1640 medium supplemented with 10% heat-inactivated fetal calf serum, 2 mM L-glutamine, 100U/ml penicillin/streptomycin at 37°C in a humidified incubator with 5% CO_2_. Cells were treated with solvent control (0.01% DMSO) or 100 μM goitrin for 72 h w/wo addition of 20 IU/ml IL-2. Supernatants were used for photometric quantification by the human TNF-alpha ELISA Ready-Set-Go kit from eBIoscience (Frankfurt, Germany) according to the manufacturer's instructions.

### DNA Isolation and PCR

DNA was isolated from 1 × 10^6^ isolated PBMC using the QIAamp DNA Blood Mini Kit (Qiagen, Hilden, Germany) according the manufacturer's protocol. A 831 bp fragment of the TAS2R38 gene was amplified using the following oligonucleotides at the indicated end concentration: forward 5′ACCAATGCCTTCGTTTTCTTGGTGA ′3 and reverse 5′ CAGCTACCAAGCCATCATCA ′3 (12). PCR reaction was performed in a total volume of 50 μl containing 20 ng DNA, 1 U Taq Polymerase (Life Technologies, Darmstadt, Germany), 0.5 μM forward primer and 0.5 μM reverse primer, dNTP mix at 0.2 mM each (Life Technologies, Darmstadt, Germany) 1.5 mM MgCl and 1x PCR-buffer (Life Technologies, Darmstadt, Germany). The PCR conditions were: 94°C for 3 min followed by 30 cycles of (94°C for 45 s, 54.2°C for 45 s, and 72°C for 90 s) and 72°C for 10 min. PCR products were cleaned up using the Monarch PCR & DNA Cleanup Kit (New England Biolabs, Frankfurt am Main, Germany) according to the manufacturer's protocol. The PCR fragment was sequenced using reverse primer PCR 5′CAGCTACCAAGCCATCATCA ′3 to analyse the SNP on position 145 of the gene, and the forward primer 5′ GGAAGGCACATGAGGACAAT′3 to analyse the SNPs on position 785 and 886 by GATC (Konstanz, Germany). The sequences were analyzed using the software Chromas 2.6.4 (Technelysium, South Brisbane, Australia). DNA concentration was analyzed using a NanoDrop (Peq lab, Erlangen, Germany). Five microliter of PCR mix were used to visualize the PCR product in agarose electrophoreses.

### Calcium Flux Assay

PBMC staining for calcium measurement was performed as described above with modifications (6). Before cell loading, isolated PBMC were washed twice with HBSS and set up at a concentration of 1 × 10^7^/ml. The staining was performed using 2 μM Fluo-4-AM and 0.02% Pluronic-127 to facilitate cell loading. After incubation in a water bath for 30 min at 37°C, cells were washed twice and resuspended in RPMI Medium 1640 with 2 % FCS. PBMC were kept in the dark for another 30 min at room temperature to allow the cells to de-esterify the Fluo-4-AM. To detect intracellular calcium release, the increase in Fluo-4-AM fluorescence was measured using flow cytometry. Each sample containing 0.5 × 10^6^ cells was analyzed for 800 s. After 60 s, cells were treated with the test substance and then measured for another 740 s. To block calcium release, cells were pre-treated with 10 μM of the phospholipase C-β2 inhibitor U73122 for 1 h prior test compound exposure. 0.1% DMSO was used as solvent control. Data were analyzed using FlowJo software (Ashland, Oregon, USA).

### Statistics

Results were analyzed using GraphPad Prism 6.0 software (La Jolla, California, USA). Data are presented as means +SD. Statistical significance was determined by the ordinary one-way ANOVA followed by Bonferroni correction test. Comparisons between two different cell types from the same individual were made by the paired *t*-test. *P* values < 0.05 (^*^) were considered statistically significant and < 0.01 (^**^) were considered highly statistically significant.

## Results

### T2R38 Expression in PBMC Subsets

**Figure 1:**
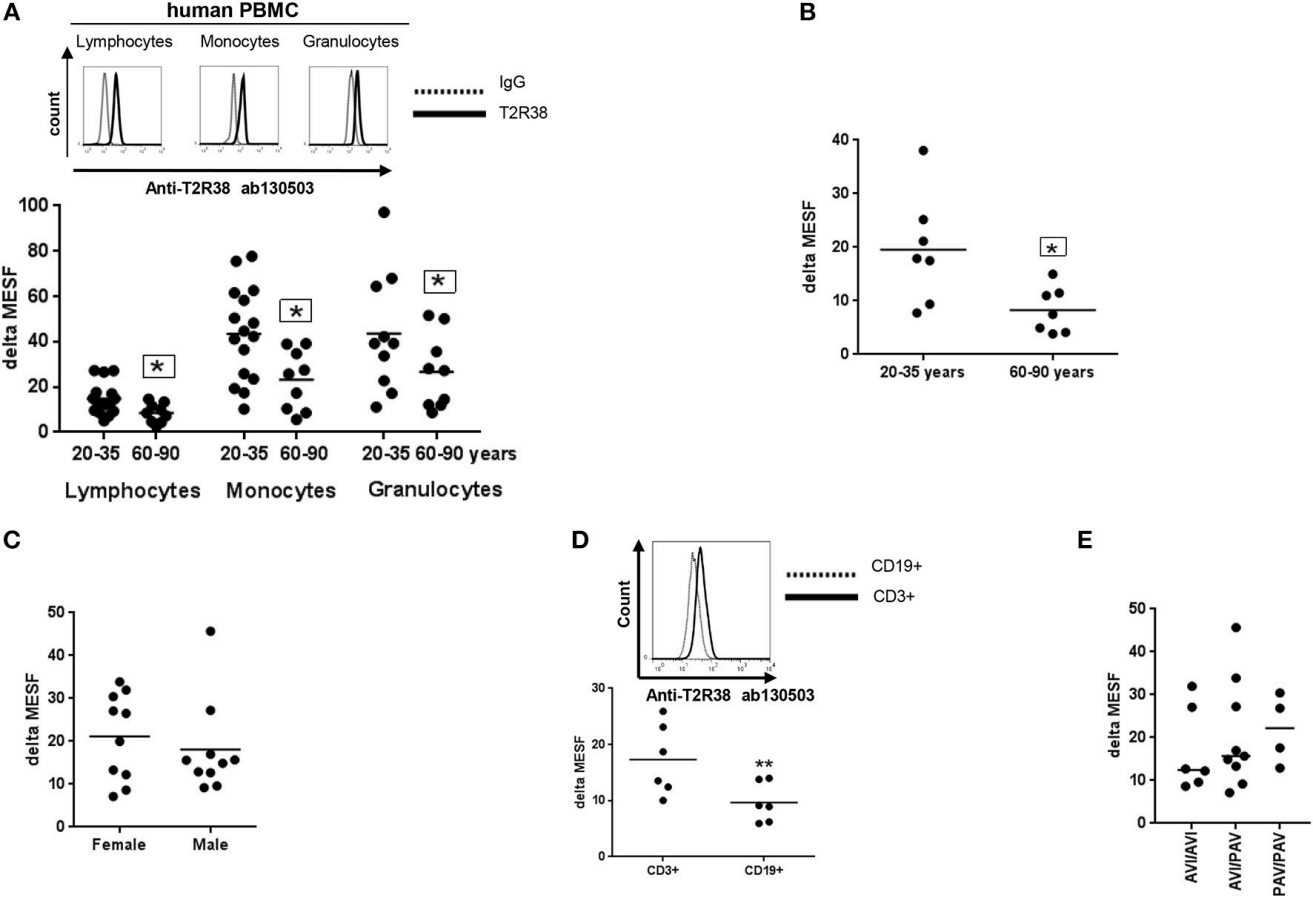
Expression of T2R38 receptor in human PBMC. **(A)** Age-related T2R38 expression in PBMC from young (20–35 years, *n* ≥ 10) and elderly (60–90 years, *n* = 9) subjects. A representative staining from one person is shown for each cell type; T2R38 (continuous line) and rabbit IgG (dotted line). **(B)** T2R38 expression in CD3+ T cells from young (*n* = 7) and elderly (*n* = 7) donors **(C)** Sex difference of T2R38 expression in lymphocytes from male and female donors (*n* = 10). **(D)** T2R38 expression in CD3-PE stained T cells and CD19-APC stained B cells from human volunteers, (*n* = 6). A representative staining from one person is shown; CD3+ (continuous line) and CD19+ (dotted line). **(E)** Haplotype analysis was done using amplification of a TAS2R38 864bp fragment followed by sequencing. PAV: **p**roline, **a**lanine and **v**aline; AVI: **a**lanine, **v**aline and **i**soleucine (*n* = 19). T2R38 expression (delta MESF) was quantified by Quantum Alexa Fluor 488 MESF using cytofluorometry (Supplementary Figure S1A), IgG was used as isotype control. Each dot presents one donor in the graphs. The gating strategy is shown in Supplementary Figure S1B. Significance of difference was calculated relatively to the respective control, *p < 0.05, **p < 0.01.

We first quantified relative T2R38 receptor expression in PBMC from healthy young and elderly donors by flow cytometry. In all experiments, Quantum™ MESF beads were used for quantification in absolute fluorescence units (see also Supplementary Figure S1A). Using this method, interlaboratory data comparison is possible and also to express not only the percentage of positive cells in a sample but also the protein level of interest in a single cell (13). Figure 1A depicts the results for T2R38 expression in lymphocytes in comparison to monocytes and neutrophils (granulocytes). A variation of 47 and 48% was seen in lymphocytes from young and elderly donors, respectively whereas the T2R38 level was significantly higher (14.75) among younger than older subjects (8.55). The level of variation for monocytes was 47 and 56.8% and for granulocytes 60.4 and 61.5%, respectively; a clear dependence on age could also be identified here for T2R38 expression in these two cell populations. We reanalyzed the data and for the population of CD3+ T-cells, the age-dependency was confirmed (Figure 1B). We also analyzed the data from lymphocytes of young donors (aged between 20 and 35) with regard to sex differences on receptor expression, but in this instance, no difference could be seen (Figure 1C). When comparing CD3+ T and CD19+ B-lymphocytes from young donors, a significant lower level of T2R38 was then found for B-cells compared to T-cells (9.66 and 17.28, respectively), Figure 1C. The hTAS2R38 gene is known to contain three single nucleotide polymorphisms (rs714598, rs1726866, rs10246939) at positions encoding amino acids 49 (encoding for either **p**roline or **a**lanine), 262 (encoding for **a**lanine or **v**aline) and 296 (encoding for **v**aline or **i**soleucine). These represent the most common variant alleles of TAS2R38, and give rise to two frequent haplotypes PAV (functional) and AVI (non-functional). The responses of the haplotypes correlate very well with individuals' bitter sensitivities (4, 14, 15) whereas for the diplotypes, the sensitivity for tasting bitter is PAV/PAV > PAV/AVI >> AVI/AVI. We investigated the most common diplotypes using sequencing analysis to determine whether there is a link between the level of receptor expression and phenotype status but this analysis failed to detect a correlation between diplotype and T2R38 expression level (Figure 1E).

### Increased T2R38 Expression Upon Activation

**Figure 2:**
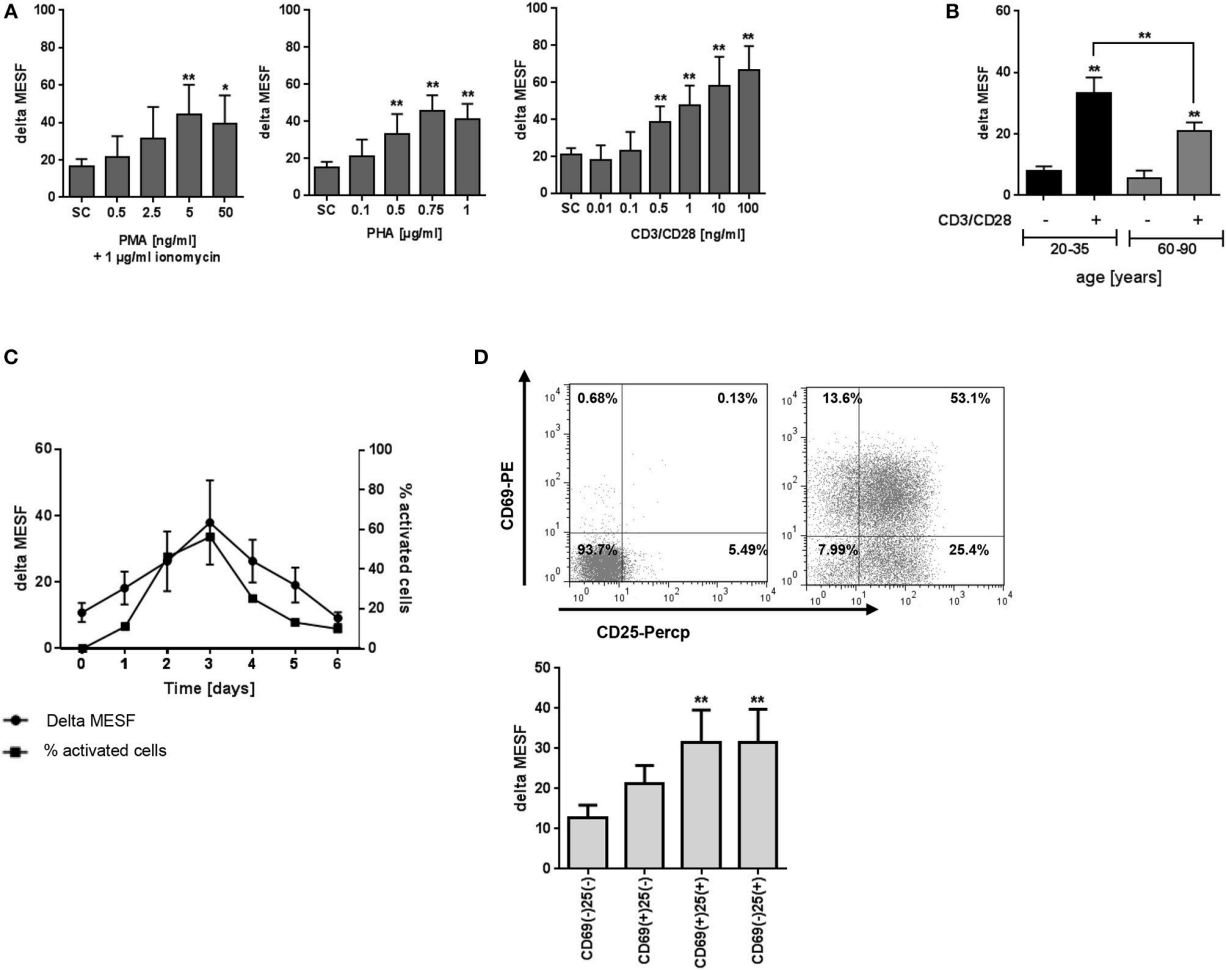
Effect of activation on T2R38 expression in PBMC. **(A)** PBMC were stimulated with different concentrations of PMA/ionomycin (1 μg/ml), PHA or anti-CD3/CD28 mAbs for 72 h (*n* ≥ 5) **(B)** Isolated PBMC from young and elderly individuals were stimulated with 1 ng/ml CD3/CD28 for 72 h (*n* ≥ 5) **(C)** PBMC were stimulated with 1 ng/ml CD3/CD28 for the indicated time points, activated cells were determined by the percentage of blast cells (*n* ≥ 3) **(D)** PBMC were stimulated with 1 ng/ml CD3/CD28 and T2R38 expression on CD69+/CD25+ CD3+T lymphocytes determined at the indicated time points (*n* = 5). A representative staining of surface markers CD69+/CD25+ at day 2 from one subject is shown as scattergram. T2R38 expression (delta MESF) was assessed by Quantum Alexa Fluor 488 MESF beads in comparison to rabbit IgG isotype control; bars are means + *SD* **(A,B)** or means ± *SD* **(C,D)**. Significance of difference was calculated relatively to the respective control, *p < 0.05; **p < 0.01. SC, solvent control. The gating strategy is shown in Supplementary Figure S2.

PBMC were next exposed to a combination of anti-CD3/anti-CD28 mAbs, PHA or PMA/ionomycin for 72 h and the levels of T2R38 expression analyzed by flow cytometry. While anti-CD3/CD28 treatment stimulates T-cells in a manner that partially mimics stimulation by antigen-presenting cells, the lectin PHA activates T-cells by binding to cell membrane glycoproteins, including the T-cell receptor (TCR)–CD3 complex (16, 17). Another non-specific activation is ionomycin, which synergizes with PMA in inducing T and B-cell activation and proliferation by direct activation of protein kinase C (16). There was a concentration-dependent increase in bitter receptor expression upon all three activation methods, whereas T-cell specific CD3/CD28 stimulation led to the strongest increase (Figure 2A). When using ionomycin/PMA activation, the maximum level of T2R38 expression was reached at 5 ng/ml PMA. For PHA activation, this was reached at 0.75 μg/ml. We further investigated the effect of CD3/CD28 activation on lymphocytes of young and elderly subjects (Figure 2B). A comparable increase in T2R38 signal was evident in both population groups upon activation of resting cells (young donors: 80.44 ± 7.8%; elderly: 77.58 ± 8.0%). Next, we studied time-dependent relations between T2R38 receptor expression and portions of the signaling molecules CD69 and CD25 expression. Freshly isolated PBMC were treated with anti-CD3/CD28 for 1 to 6 days. When comparing the time course of T2R38 expression and the blast transformation it was found that the level of T2R38 marker was lower in small lymphocytes than in the large blast cells (Figure 2C and Supplementary Figure S2). An increased T2R38 level was detectable within 24 h after CD3/CD28 addition (1.66-fold compared to 0 h). Peak expression of T2R38 was reached after 72 h (3.38-fold), and then subsequently decreased. We also analyzed the level of T2R38 in relation to the expression of activation markers on the surface of PBMC after 48 h of stimulation. Compared to cells stained negative for the very early activation marker CD69 and the late activation marker CD25, the amount of T2R38 was increased in CD69+CD25- cells. The level further increased in CD69+CD25+ cells which was then comparable to CD69-CD25+ cells (Figure 2D).

### T2R38 Expression in CD4+ and CD8+ T Cells

**Figure 3:**
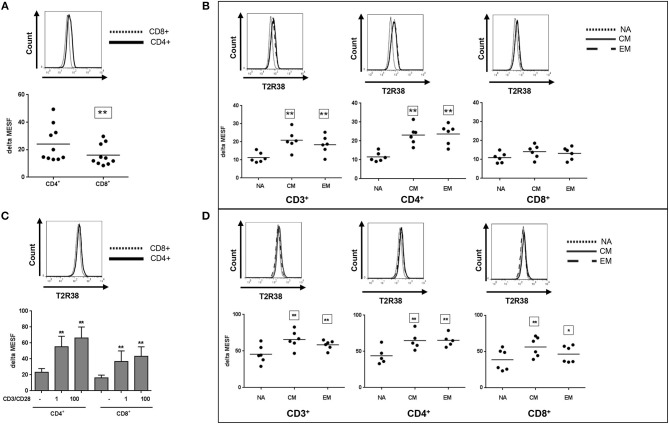
T2R38 expression status in T cell subsets. T2R38 expression was quantified in **(A,B)** freshly isolated cells (*n* ≥ 6) or **(C,D)** CD3/CD28-stimulated cells after 72 h (*n* ≥ 5). Each dot represents one donor, bars are means + *SD*. NA, naïve, CM, central memory, EM, effector memory cells. Representative histograms of different stains from one donor are shown. The gating strategy is given in Supplementary Figure S3. T2R38 expression (delta MESF) was assessed by Quantum Alexa Fluor 488 MESF beads in comparison to rabbit IgG isotype control. Significance of difference was calculated relatively to the respective control, *p < 0.05, **p < 0.01.

In Figure 3, the T2R38 expression is shown in CD4+ helper/inducer cells and CD8+ cytotoxic/suppressor cells from freshly isolated PBMC. Both subpopulations were positive for T2R38, however, the level of expression was different; the CD4+ subpopulation expressed significant higher levels (24.12) as compared to CD8+ cells (16.07) (Figure 3A). In immunity, T-cell differentiation and memory and effector T-cells play an important role against pathogens (18). Thus, we further investigated T2R38 expression in these subsets using CD45Ro and CD62L markers for discrimination. For both CD3+ as well as CD4+ T cells, the naïve (NA) cells expressed significant lower levels of T2R38 protein (in range of 0.5-fold) compared to central memory (CM) or effector memory (EM) cells (Figure 3B). Upon activation by anti-CD3/CD28, T2R38 protein upregulation was seen in both CD4+ and CD8+ subpopulations (Figure 3C) and again, bitter receptor expression was significantly lower for the NA cell population compared to CM or EM cells in CD3+, CD4+, and CD8+ cells (Figure 3D).

### Calcium Influx and TNF-Alpha Secretion

**Figure 4:**
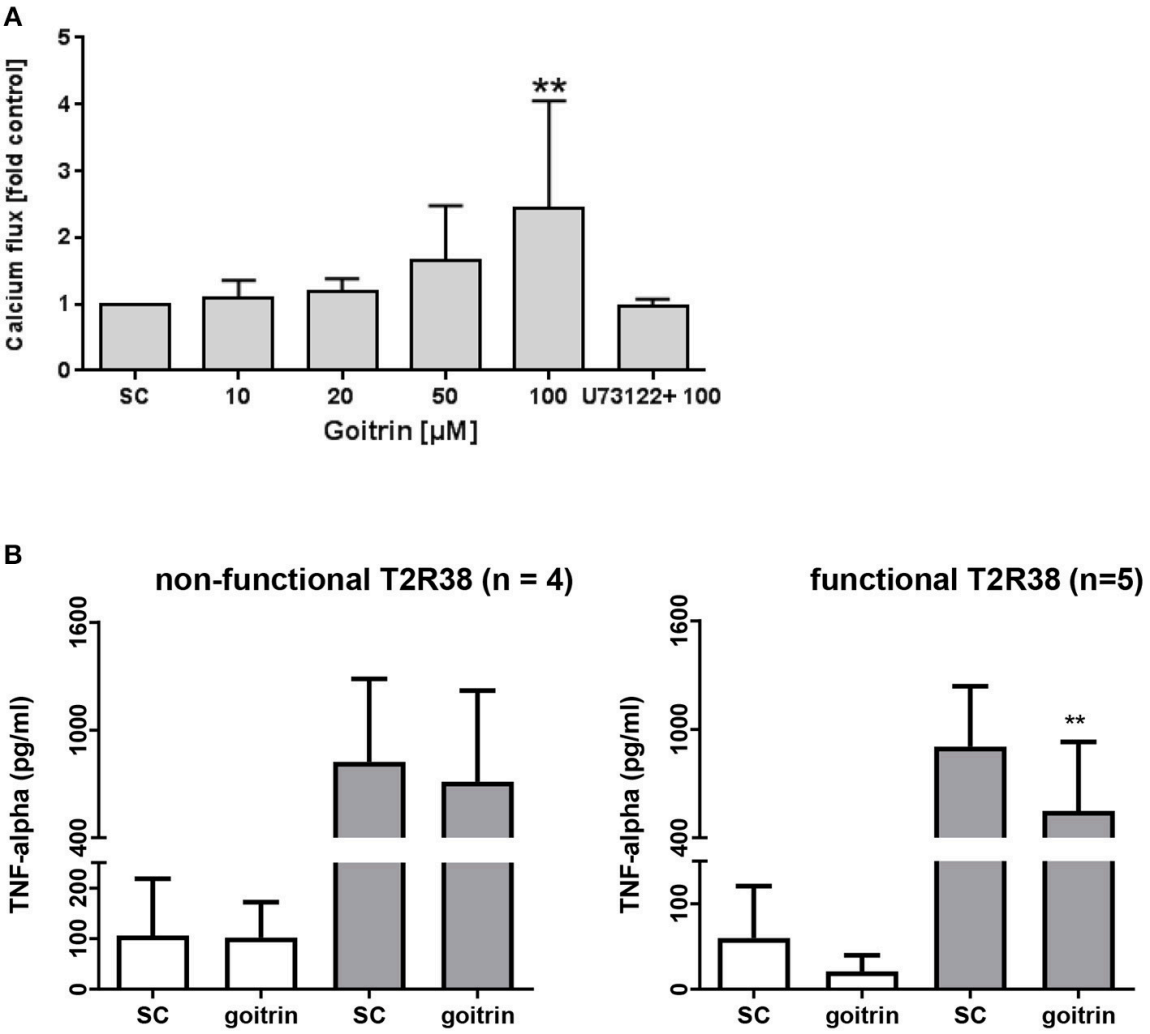
Analysis of calcium flux and TNF-alpha secretion. **(A)** FACS-based measurement of calcium flux was detected in lymphocytes (PAV haplotype) probed with Fluor-4 AM (F_EM:516nm)_ after treatment with different concentrations of goitrin alone or 1 h pre-incubation with 10 μM PLC-β2 inhibitor U73122. Baseline fluorescence was recorded for 60 s before addition of goitrin and fluorescence was measured for a total of 800 s. Calcium response was calculated as the ratio of the maximum peak post stimulation to basal level using FlowJo software. The gating strategy is shown in Supplementary Figure S4. Bars are means + *SD, n* ≥ 8. Significance of difference was calculated relatively to the respective control, *p < 0.05. (**B)** PBMC with functional (PAV haplotype) and non-functional (AVI/AVI) T2R38 receptor were exposed to 100 μM goitrin or 0.01% DMSO for 72 h w/wo IL-2 and TNF-alpha secretion was analyzed using an ELISA kit. Results are mean values + *SD*, **p < 0.01. Significance of the difference was determined compared to the respective solvent control (SC, 0.01% DMSO).

Calcium influx is a key signaling process that controls a wide array of T-cell functions. Thus, we analyzed the effect of the natural T2R38 agonist goitrin in human PBMC from functional T2R38 (PAV haplotype) donors using flow cytometry. Calcium flux was detected in lymphocytes probed with Fluo-4 AM (FEm: 516 nm) upon stimulation with goitrin in a concentration-dependent manner, starting at a concentration >20 μM (Figure 4A). At 100 μM goitrin, calcium flux was increased by 144% as compared to the solvent control. Cell pretreatment with the specific PLCβ-2 inhibitor U-73122 before addition of goitrin at 100 μM completely blocked calcium flux (Figure 4A). We then tested the impact of goitrin on secretion of the potent proinflammatory cytokine TNF-alpha (Figure 4B). Using PBMC with functional (PAV haplotype) or non-functional (AVI/AVI diplotype) T2R38 receptor, a differential response to goitrin exposure was observed. Whereas, goitrin did not affect TNF-alpha secretion in samples with non-functional T2R38, a significant inhibition of TNF-alpha release could be detected upon goitrin exposure in samples from functional T2R38 as compared to the solvent control.

## Discussion

To date, the expression pattern of T2R38 bitter receptor in human lymphocytes and their subsets is not documented and here we demonstrate that T2R38 is differentially expressed among lymphocyte populations. In contrast to CD3+ T-cells, CD19+ B-cells had a significantly lower expression of T2R38. Moreover, B-cells had the lowest level of T2R38 compared to any other cell type examined, which might indicate a minor role for this bitter receptor on B-cells. A hallmark of the adaptive immune response is T-cell activation, which involves two major steps of signal transduction events. TCR complex, upon specific antigen recognition, initiates a cascade of intracellular signaling events which ultimately result in T-cell proliferation and differentiation into effector cells; a critical arm of the adaptive immune response (19). Thus, establishing knowledge on the regulation of T2R38 expression downstream of TCR is of vital importance for insight into the potential role of the bitter receptor in the immune response by T-cells. We present new evidence that under physiological conditions of human T-cell stimulation, induction of T2R38 is timely regulated and follows activation. After T-cell activation, several surface markers are upregulated, each at a different stage of the activation process. CD69 is regarded as the earliest activation marker on T-cells (20). CD69 peak elevation precedes the appearance of CD25 that is considered the most prominent cellular activation marker and plays a key role in IL-2 responsiveness, finally resulting in the full expression of immune responses (21). We demonstrate here that the T2R38 level increased in the population of CD69+ and further CD25+ cells which suggests that T-cell activation and differentiation may alter the responsiveness to bitter receptor agonists.

Unlike CD4+ cells, we found that CD8+ cells express lower levels of T2R38 which could indicate altered capacity of CD8+ cells to respond to bitter compounds. Similar results were obtained with activation of these subsets with antiCD3/CD28 mAbs.

EM cells display immediate effector function and migrate to inflamed peripheral tissues to control the initial pathogen exposure. CM cells are functionally characterized by their increased proliferation potential after antigen reencounter, seeding the peripheral tissues with new effector cells after stimulation (19). In our study, we showed that T2R38 expression is already elevated in mature/differentiated T-cells at quiescence state. Upon stimulation, the T2R38 protein level further increased in EM as well as CM cells compared to the naïve population, which is another indicator that the bitter receptor could play a role in the pathogen response of the adaptive immune system.

The T2R38 receptor was detected in all cell samples but within one age group broad inter-individual differences in the absolute amount of T2R38 receptor in a single cell were observed. Using buffy coats from the Bavarian Red Cross Blood Bank (Germany), in one study from Malki et al. (22) T2R38 mRNA expression was shown at 21–40% of the reference in CD14+ monocytes or CD3+ lymphocytes which was lower compared to most of the other T2Rs under investigation. Several sensory studies in humans also demonstrated a broad range of bitter taste perception, even within the same haplotype (4, 23, 24). Further, taste sensitivity changes were reported with age. A study conducted by the National Institutes of Health (NIH) on 2557 subjects ranging from 12 to 85 years concluded that the intensity of bitterness decreases with subjects' age (25). In particular, the psychophysical bitter sensitivities as recognized by the T2R38 receptor on the tongue also have been shown to decrease over lifespan (26). In another study by Mennella and group on children and their mothers, this observation was further confirmed (27). Whissell-Buechy and Wills (28) reported that sensitivity to the T2R38 agonist phenylthiouracil also declines with age. Whether the loss in bitter taste sensitivity in older subjects is indeed due to a change in T2R38 receptor expression on the gustatory cells of the tongue remains to be determined. It is of note that in PBMC subsets, we also observed an age-dependency in T2R38 receptor expression with significant lower T2R38 levels in cells from the elderly. So, it would be interesting to find out if progressive aging could be linked with a general decrease in T2R38 receptor expression in tissues. Possibly, a lower T2R38 level in immune cells of the elderly could add to an impaired defensive capability of the adaptive immune system that is observed with increasing age (29, 30). Here, loss of cell surface molecules, as e.g. the CD28 receptor, is a hallmark for aged cells which in consequence results in multiple changes in gene expression. On the other hand, it is known that the signaling of agonist stimulation by human GPCR, such as the T2R38, is amplified and thus high levels of expression are not absolutely required to reach relevant physiological effects (31).

So far, much has been done to understand the signal transduction mechanisms of bitter taste receptors and much progress has been achieved, although it is still not fully understood. Basically, binding of an T2R agonist results in activation of heterotrimeric G protein activation which in turn triggers its dissociation into Gα gustucin and Gβ3/Gγ13 subunits. The subunits then subsequently activate phospholipase C-β2 (PLCβ-2), which hydrolyses phosphatidylinositol-4,5-bisphosphosphate (PIP2) to inositol-1,4,5-triphosphase (IP3) and diacyl glycerol (DAG). IP3 stimulates Calcium release from intracellular stores by acting on IP3 receptors on the endoplasmatic reticulum (32). In human lymphocytes, Calcium signaling can ultimately result in cell proliferation and cytokine production (33). In our experiments, the natural T2R38 agonist, goitrin, indeed triggered Calcium release at concentrations >20 μM in human lymphocytes from taster (PAV haplotype) volunteers. Using the PLCβ-2 inhibitor U-73122, we could further show that the Calcium mobilization predominantly took place through the goitrin-T2R-Gα*βγ*-PLC pathway which provides functional relevance for T2R38 receptor expression in human lymphocytes.

We could additionally show that goitrin selectively triggers an anti-inflammatory response in terms of reducing TNF-alpha secretion in PBMC with functional but not non-functional T2R38 receptor. Goitrin is one of the most abundant glucosinolate-myrosinase products in *Brassicaceae* (34). It is known to be toxic in terms of interfering with thyroid hormone synthesis and consequent iodine uptake due to its potent thyroid peroxidase inhibition capacity (35). A highly sensitive biosensor, which utilized the T2R38 protein coupled with a carboxylated polypyrrole nanotube-field effect transistor, responded to goitrin at a concentration of <1 μM (36). However, in human volunteers, the threshold for goitrin tasting has been reported much higher with a range of concentrations between 64 and 4,096 μM (37). The same group used a functional assay with T2R38 overexpressing HEK293T cells to demonstrate that goitrin triggered a response at >10 μM, which fits well with our observations in lymphocytes. To date, no data for human plasma levels of goitrin after consumption of *Brassicaceae* are available. Thus, it is currently unknown whether goitrin comes into contact with cells of the adaptive immune system in the concentrations necessary for receptor activation, which is also true for other natural T2R38 receptor ligands.

In summary, the present data demonstrate expression of the bitter receptor T2R38 in resting and activated human lymphocytes, and the findings suggest a relevance of this extra-gustatory T2R38 expression for human immunity. However, studies of more depth are needed including the confirmation of T2R38 expression dynamics on mRNA level. The role of T2R38 in extra-oral tissues is still not very clear. A role in local host defense has been proposed in earlier studies (11). Further, non-functional diplotypes are associated with increased susceptibility to chronic rhinosinusitis (5), more severe sinonasal symptoms when suffering from cystic fibrosis (38), increased risk for dental caries (39), increased risk for colorectal cancer (40), and gastric cancer (41). Findings by Orsmark-Pietras et al. (42) indicate that bitter taste receptors might mediate an anti-inflammatory effect. In their study, they used the T2R agonists chloroquine and denatonium to modulate lipopolysaccharide-induced pro-inflammatory cytokine and prostaglandin (PG)E_2_ release in human leucocytes from patients. Many natural bitter tasting compounds including ITC are prominent multi-target compounds interacting with a broad number of signaling pathways and molecules (43, 44). They are well-known for their anti-inflammatory, immune modulatory potential and intensively investigated as cancer preventive agents for the general population (45–49). To date, for allyl ITC, a selective response to the functional variant of T2R38 has been demonstrated *in vitro* using HEK293T cells (50). At present, one can only speculate whether the reported immune modulation of these natural compounds is indeed directly linked with T2R38 interaction. The results derived here with goitrin argue for a genotype-specific anti-inflammatory response in human immune cells. If this finds further confirmation, the consequences for non-functional genotype carriers will need to be explicitly addressed.

## Author Contributions

EL designed the study. HT, PR, CH, and RS designed and carried out the experiments. HT, CH, PR, RS, and EL prepared the graphs and analyzed the data. EL and HT wrote the paper. All authors reviewed and approved the paper.

### Conflict of Interest Statement

The authors declare that the research was conducted in the absence of any commercial or financial relationships that could be construed as a potential conflict of interest.
