# Supplementary material for: Human T2R38 Bitter Taste Receptor Expression in Resting and Activated Lymphocytes
Source: Front Immunol. 2018 Dec 11;9:2949. doi: 10.3389/fimmu.2018.02949 (PMC6297872; doi:10.3389/fimmu.2018.02949)

Supplementary Material

Human T2R38 bitter taste receptor expression in resting and activated lymphocytes

Hoai TT Tran[1,2], Corinna Herz[1], Patrick Ruf [1] , Rebecca Stetter[1] and Evelyn Lamy[1*]

[1]Molecular Preventive Medicine, University Medical Center and Faculty of Medicine – University of Freiburg, Freiburg, Germany
[2]Pharmaceutical Bioinformatics, Institute of Pharmaceutical Sciences, Albert-Ludwigs-University, Freiburg, Germany

* Correspondence:
Dr. Evelyn Lamy
Evelyn.lamy@uniklinik-freiburg.de

Supplementary Figure Legends

Supplementary Figure S1:

(A) Gating of MESF calibration beads used for calibration curve; numbers indicate four different bead populations. **(B)** Scattergrams showing gating strategy and frequency of granulocytes/neutrophils (CD11b+), monocytes (CD14+), T lymphocytes (CD3+) and B lymphocytes (CD19+)

Supplementary Figure S2:

PBMC were stained with appropriate markers at different time points (day 0-6) as described in Material and Methods. An FSC/SSC-plot was made and all lymphocytes were gated. The lymphocyte population was copied to an SSC/CD3-scatterplot identifying T-cells (CD3+), followed by histograms of CD69+ and CD25+ cells for analyzing activated T lymphocytes at day 0 to day 3.

Supplementary Figure S3:

CD45RO/CD62L staining was used for determining naïve, central memory or effector memory cells of lymphocyte subsets (CD3/CD4/CD8) at day 0 or day 3.

Supplementary Figure S4:

An FSC/SSC-plot was made and all lymphocytes were gated. The lymphocyte population was copied to an FL1-Fluor-4/Time -scatterplot identifying Calcium flux response to goitrin. Calcium response was calculated as the ratio of the maximum peak post stimulation to basal level using FlowJo software. 100ng/ml ionomycin was used as positive control.

Supplementary Figure S1

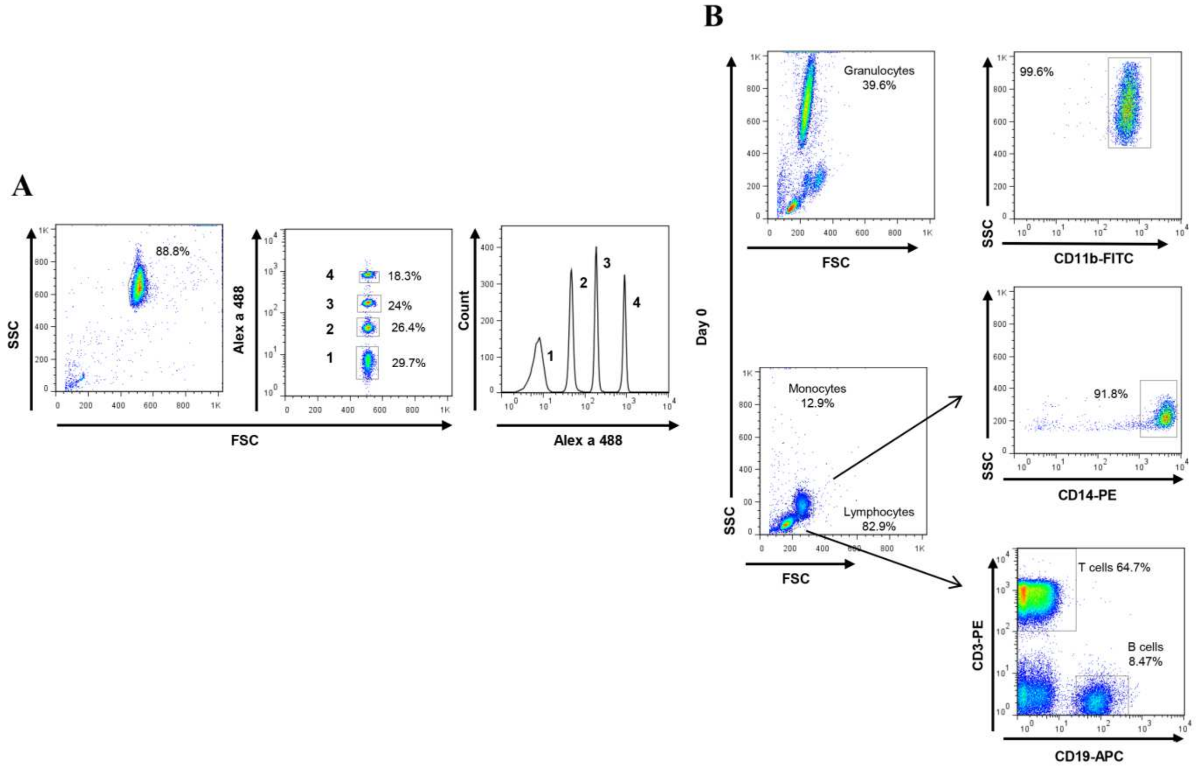

Supplementary Figure S2

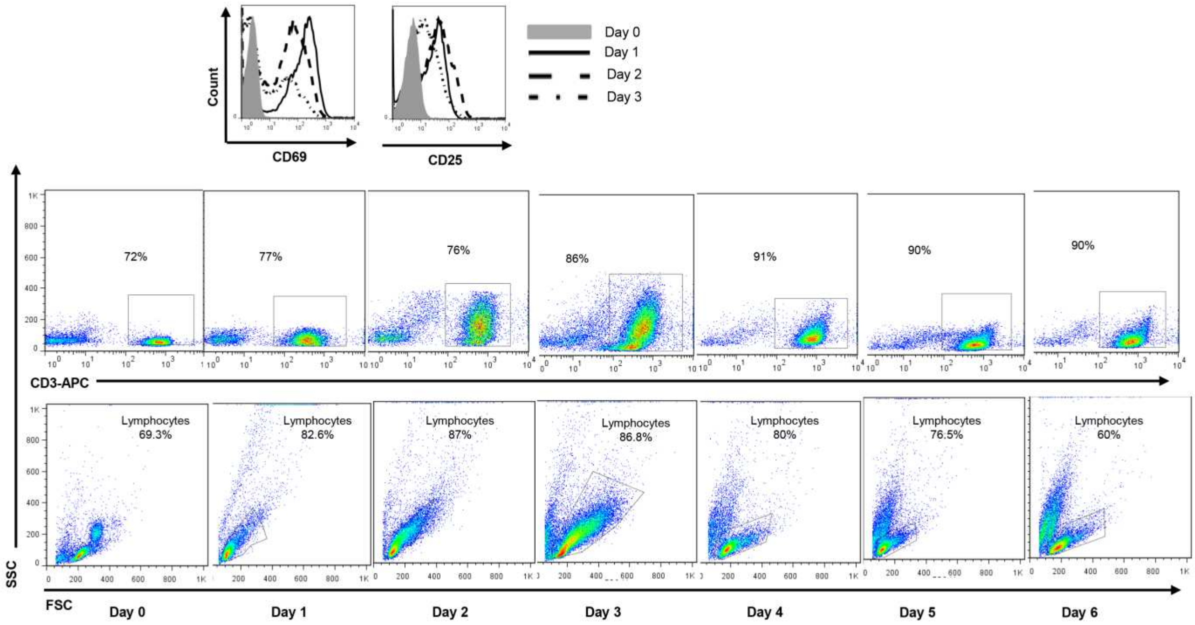

Supplementary Figure S3

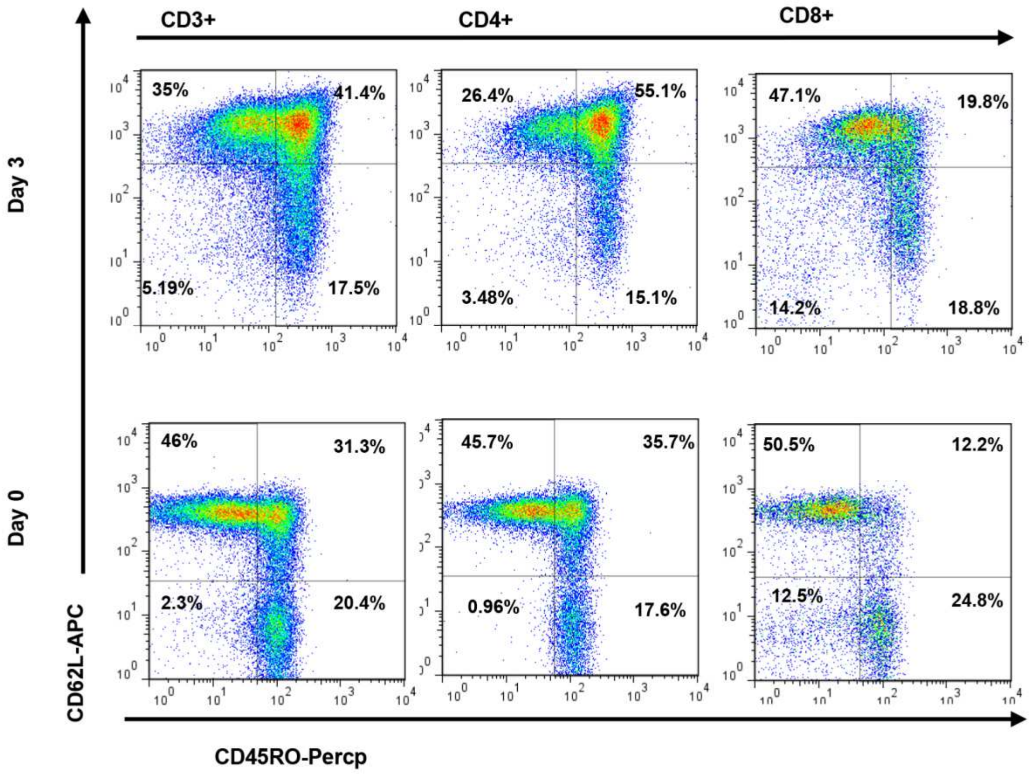

Supplementary Figure S4

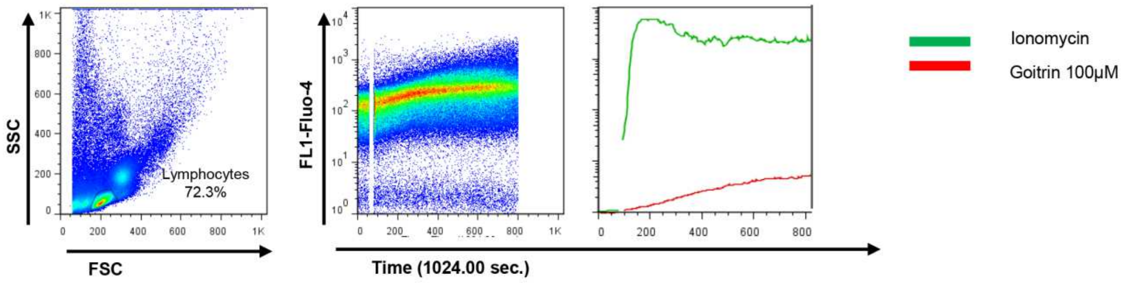

Supplement: Supplementary file 1 [file Presentation_1.PDF]
